# Sexual and reproductive health information needs; an inquiry from the lens of in-school adolescents in Ebonyi State, Southeast Nigeria

**DOI:** 10.1186/s12889-024-18584-w

**Published:** 2024-04-22

**Authors:** Ozioma Agu, Ifunanya Clara Agu, Godstime Eigbiremolen, Ifeyinwa Akamike, Chinyere Okeke, Chinyere Mbachu, Obinna Onwujekwe

**Affiliations:** 1 Health Policy Research Group, University of Nigeria Enugu Campus, Enugu, Nigeria; 2 Department of Community Medicine, University of Nigeria Enugu Campus, Enugu, Nigeria; 3 Department of Economics, University of Nigeria Nsukka, Nsukka, Nigeria; 4 Department of Community Medicine, Alex Ekwueme Federal University Teaching Hospital, Abakaliki, Ebonyi State Nigeria; 5 Department of Health Administration and Management, University of Nigeria Enugu Campus, Enugu, Nigeria

**Keywords:** Adolescents, Information needs, Predictors, Sexual and reproductive health

## Abstract

**Background:**

It is important to understand the sexual and reproductive health (SRH) needs of adolescents from the adolescents themselves to address their needs properly. Hence, this paper provides new knowledge on the information needs on SRH among adolescent boys and girls in selected secondary schools in Ebonyi state, southeast Nigeria.

**Method:**

A comparative assessment was conducted among adolescent boys and girls in public secondary schools that received a specific school-based SRH intervention (group A) and those that did not receive the intervention (group B). These schools were spread across six urban and rural local government areas in Ebonyi state, southeast Nigeria. A structured interviewer-administered questionnaire was used to collect data from 514 adolescents aged 13 to 18 on their stated needs for SRH information and services. Categorical variables were compared using the Chi-square test, and predictors were determined using logistic regression analysis. The statistical significance was determined at *p* < 0.05.

**Result:**

Majority of the adolescents (82% of intervention group and 92% of non-intervention group) identified puberty and pubertal changes as perceived SRH information need for adolescents (χ2 = 7.94; p-value = 0.01). Adolescents who received SRH intervention have 3.13 (*p* < 0.001) times the odds of perceiving the need for adolescents to be provided with SRH information than adolescents who did not receive SRH intervention. The odds of perceiving the need for adolescents to be provided with SRH information for adolescents who reside in urban communities are 0.31 (*p* < 0.001) times the odds for adolescents who resides in rural communities. That is, the perception odds are higher adolescents who reside in rural communities. Multivariate regression of specific SRH information showed the location of residence as a strong predictor of adolescents’ perceived need for information on ‘puberty and pubertal changes’ (OR = 0.30; *p* = 0.001), ‘safe sex and sexual relations’ (OR = 0.33; *p* < 0.001) and ‘prevention of pregnancy and use of contraceptives’ (OR = 0.28; p < 0.001). Adolescents in senior secondary school have 2.21 (p = 0.002) times the odds of perceiving the need for adolescents to be provided with specific SRH information than adolescents who are in junior secondary school.

**Conclusion:**

Adolescents’ age, location of residence, and study group were found to be strong predictors of SRH information needs. This suggests the need for in-school adolescents to be provided with substantial and continuous SRH information for healthy living and making informed SRH choices. In developing SRH interventions that will achieve optimal effectiveness in the lives of adolescents in school, different demographic factors should be considered for context-specific and appropriate strategies.

**Supplementary Information:**

The online version contains supplementary material available at 10.1186/s12889-024-18584-w.

## Background

Globally, adolescents are developing in an environment of active change that either enables them to thrive or compels them to struggle for their existence [1]. Literature shows that although adolescents are at higher risk for sexual and reproductive health (SRH) problems [2, 3], they still experience limited access to life-saving health information and services [4]. This is particularly so in developing countries such as Nigeria where cultural norms, ideologies and overall lack of attention to adolescent health constrains the provision of comprehensive SRH information and services [5–7].

Efforts have been made to ensure that comprehensive SRH education is provided in schools to advance gender equality and reduce risky sexual adolescents’ behaviors [8, 9]. Specifically, In Nigeria, a guide has been designed to help schoolteachers provide information and skills students need to make healthy decisions, and respect and value themselves [9]. However, the implementation is opposed by conflicting gender-based perceptions, lack of adequate skills and knowledge, cultural norms, and social values [8]. Also, a Nigerian study has shown that adolescents who seek SRH services are either denied certain SRH services, treated unfairly, shamed, or judged which further limits their access to SRHR information and services [10].

In Nigeria, about 26.1% of the population is within 12-24years [11]. The prevalence of sexually transmitted infections is high among adolescents, with about 40% of new HIV infections occurring in this age group [12–14]. Moreover, a good proportion of adolescents experience unwanted pregnancies, unsafe abortions, and resultant consequences and complications [15, 16]. Adolescents need to be provided with SRH information, education, and services as it helps them to understand their sexual health and protect them from being victims of negative SRH outcomes such as unplanned pregnancies [17].

Notwithstanding the many SRH challenges that adolescents face, their access to SRH information and services is poor and uptake of available youth-friendly health services is poor [18]. Although demographic factors such as gender, age, schooling, income, living status, and psycho-cultural and health system factors have been implicated in the uptake of SRH services by adolescents [18–21], there is less clarity on whether these services are meeting the needs of adolescents. It is therefore important to underline the SRH needs of adolescents from their perspectives, to understand how the available SRH services could be better aligned to meet their needs.

This paper provides new knowledge on the information needs on sexual and reproductive health among adolescent boys and girls in selected secondary schools in Ebonyi state, southeast Nigeria. To understand the differences in adolescents’ need for SRH information, schools that have received SRH interventions and non-intervention schools were selected for this study. The information will be useful to program managers and implementers to design context-specific interventions that target the provision of SRH information to adolescents in schools.

## Methods

### Study design and study area

A comparative quantitative assessment was carried out among students in six (6) public (government) secondary schools that received a school based SRH intervention (the intervention group) and six public secondary schools that did not receive the intervention (the non-intervention or control group). There was no baseline assessment of the SRH needs of the students in both groups of schools before the intervention was implemented. The baseline study focused on collecting community level data from households because the original plan was to implement a community campaign for adolescents and their parents or guardians. However, following the COVID-19 pandemic and restrictions to social gatherings, a school-based intervention was designed and implemented in the government secondary school within the community. The control secondary schools for the comparative assessment were purposively selected from the same local government area as the schools that received the intervention. The researchers ensured that both schools matched in terms of ownership (government-owned) and the sexes of students that are admitted.

The schools were spread across six urban and rural local government areas (LGAs) in Ebonyi state, southeast Nigeria. Ebonyi State has thirteen LGAs that are grouped into six senatorial/geopolitical zones. The characteristics of the secondary schools in terms of location, education system, and whether they received the targeted school-based intervention, are found in supplementary file1.

### Description of the school-based intervention

The school-based interventions were co-designed and implemented by a team of researchers from University of Nigeria, and school health focal officers from State Ministry of Health, State Ministry of Education and State Ministry of Information, Ebonyi State [22]. The study was registered (retrospectively) with ISRCTN registry, with study registration number ISRCTN29711792.

The interventions were implemented over a period of eighteen months. The intervention was multi-faceted, comprising (i) a training workshop of 29 state trainers to raise a critical mass of competent and skilled trainers to train other providers, (ii) three-day training of 22 school teachers (including principals and guidance counselors), (iii) two-day training of 22 peer mentors (iv) establishment and inauguration of school-based youth health clubs, (v) distribution of SRH customized educational items- notepads, fliers, shirts, caps, wrist bands, pens), vi). Supportive supervision, and vii) community sensitization and engagement of 20 community gatekeepers from each selected community.

The participants in the school-based training included the school principals, biology teachers, health education teachers, guidance counselors (G&C), and senior secondary students purposively selected from six government schools located in the participating communities. The training was delivered using a manual adapted from the national guideline and modified to suit the context. The manual consists of eight modules: (i) Introduction to adolescence and adolescent health; (ii) sexuality and sexual behaviours; (iii) sexually transmitted infections; (iv) principles and practice of Counseling; (v) Pregnancy and prevention of pregnancy; (vi) counseling practices on selected health issues of adolescents; (vii) optimal adolescent and youth-friendly services; and (viii) record-keeping and health information systems and (ix) principles and practice of counselling. The training was facilitated by five researchers and two boundary partners and delivered using multiple formats - PowerPoint presentations, flip charts, demonstration, roleplay, and discussion. These interventions are described in detail in a published article [23].

### Study population, sample size, and sampling technique

The study population comprised of in-school adolescent boys and girls who were between 13 and 18 years of age in each of the public schools selected from the six study LGAs.

The sample size was determined using the formula for two populations (intervention group and non-intervention group) [24]. Assuming a 95% confidence interval, 80% power, observed difference of 55%, and a 10% non-response rate, a minimum sample size of 458 was estimated. To check for the robustness of data and incomplete data or errors, the minimum sample size of 458 was exceeded during data collection.

A multi-stage sampling method was used to recruit participants for the study. In the first stage, two LGAs were purposively selected from the three existing senatorial zones in the State to ensure geopolitical and geographical (urban/rural) representation. These LGAs were listed by the State government as having the highest rates of unwanted adolescent pregnancy. The second stage involved the purposive selection of two communities from each selected LGA. In each LGA, a community where adolescent SRH intervention has been implemented and another community without any intervention were selected based on the availability of a functional youth friendly SRH primary healthcare (PHC) facility and the need for SRH intervention. In the third stage, a public secondary school was randomly selected for the study in each community.

A total of 514 in-school adolescents were selected and interviewed from the twelve schools, giving an average of 42 to 43 students per school. The students were selected from the class register through a simple random sampling technique. Numbers were proportionately allocated to the six levels of study (junior and senior), giving 8–9 students per class. Using a table of random numbers generated for each class, students were selected from each level till the desired sample size was achieved.

### Data collection

This study adapted the WHO illustrative questionnaire for interview surveys with young people [25]. Forty-two (42) research assistants were recruited and trained for four days to assist with the data collection. The research assistants were paired to collect data from respondents in November 2021 for 14 days (excluding Sundays) using paper and electronic copies of the questionnaire. Individual matching of information in the paper and electronic copies of each questionnaire was carried out before the data were uploaded to the central server to ensure good data quality and accuracy.

### Data analysis

To examine the perceived needs for sexual and reproductive health (SRH) information and services among in-school adolescents, we employed both descriptive and logistic regression analysis using STATA statistical software. For descriptive statistics, data were summarized using frequency and percentage for categorical variables and means and standard deviations were reported for non-categorical variables. The chi-square test was used to compare differences in the sociodemographic characteristics and the outcome variables (SRH information needs) across the two groups of respondents. The statistical significance level determined by a *p*-value of < 0.05 was reported.

The variables comprised the independent and dependent/outcome variables. The independent variables comprised study groups (intervention or non-intervention), gender, level of education, location of residence (whether urban or rural), age, whether an individual works for pay and the socio-economic status of the individual’s family. The outcome variable comprised information needed on, (i) puberty and pubertal changes, (ii) safe sex and sexual relations, (iii) use of condoms, (iv) prevention of STIs, (v) sexual violence and rape, and (vi) prevention of pregnancy and contraceptive use.

To determine the predictors of the perceived need for each specific SRH information need, a multivariate regression was performed (Tables 1 and 2). The dependent variables were (i) puberty and pubertal changes, (ii) safe sex and sexual relations, (iii) use of condoms, (iv) prevention of STIs, (v) sexual violence and rape, and (vi) prevention of pregnancy and contraceptive use. To assess each specific SRH information need of in-school adolescents, individual responses were given scores; “1” for ‘yes’ (perceived SRH information need) and “0” for ‘No’ (SRH information perceived as not needed).

**Table 1:** Logistic regression of adolescents’ perceived need for specific SRH information that should be provided

Variables	Puberty and pubertal changes			Safe sex and sexual relation			prevention of pregnancy and contraceptive use		
	OR	p-value	95%CI	OR	p-value	95%CI	OR	p-value	95%CI
***Study group*** *(non-intervention)*									
*Intervention group*	0.67	0.359	0.28–1.59	2.90	< 0.001*	1.07–5.07	3.76	< 0.001*	1.82–7.79
***Gender*** *(male)*									
*Female*	1.27	0.487	0.65–2.51	1.12	0.639	0.70–1.78	1.82	0.055	0.99–3.35
***Age***	1.02	0.064	1.00-1.04	1.02	0.002*	1.00-1.03	1.04	< 0.001*	1.02–1.06
***Educational Level*** *(Junior secondary)*									
*Senior Secondary*	0.69	0.282	0.35–1.36	1.37	0.157	0.89–2.12	1.70	0.070	0.96–3.03
***Location of*** **residence** *(Rural)*									
*Urban*	0.30	0.001*	0.15–0.63	0.33	< 0.001*	0.21–0.54	0.28	< 0.001*	0.15–0.50
***Work for pay*** *(No)*									
*Yes*	0.79	0.612	0.03–1.95	1.66	0.101	0.91–3.04	1.65	0.165	0.81–3.33
***Socio-economic status***	0.10	0.993	0.73–1.37	0.10	0.994	0.81–1.23	1.00	0.979	0.76–1.30

* Statistical significance (p-value < 0.05)

*N* = 408

**Table 2:** Logistic regression of adolescents’ perceived need for specific SRH information that should be provided

Variables	Use of condom			Sexual violence and rape			Prevention of STIs/RTIs		
	OR	p-value	95%CI	OR	p-value	95%CI	OR	p-value	95%CI
***Study group*** *(non-intervention group)*									
*Intervention group*	*2.98*	< 0.001*	*1.63–5.47*	*3.31*	< 0.001*	*1.72–6.38*	*1.65*	*0.079*	*0.94–2.87*
***Gender*** *(male)*									
*Female*	*0.92*	*0.745*	*0.56–1.51*	*1.28*	*0.355*	*0.76–2.18*	*1.40*	*0.177*	*0.86–2.27*
***Age***	*1.03*	< 0.001*	*1.02–1.05*	*1.03*	< 0.001*	*1.02–1.05*	*1.03*	< 0.001*	*1.01–1.04*
***Educational Level*** *(Junior secondary)*									
*Senior Secondary*	*2.21*	0.002*	*1.35–3.62*	*1.62*	*0.067*	*0.97–2.71*	*0 0.82*	*0.377*	*0.52–1.28*
***Location of*** **residence** *(Rural)*									
*Urban*	*0 0.29*	< 0.001*	*0.17–0.48*	*0.49*	0.008*	*0.29–0.83*	*0 0.31*	< 0.001*	*0.19–0.51*
***Work for pay*** *(No)*									
*Yes*	*1.06*	*0.864*	*0.56–1.98*	*1.35*	*0.373*	*0.70–2.60*	*0.87*	*0.641*	*0.48–1.56*
***Socio-economic status***	*1.16*	*0.208*	*0.92–1.46*	*1.21*	*0.134*	*0.94–1.54*	*1.23*	*0.071*	*0.98–1.53*

*Statistical significance (p-value-< 0.05)

*N* = 408

In Table 3, a composite score of the six outcome variables was generated and further categorized to measure adolescents’ perceived need for SRH information. A binary logistic regression was performed, and odds ratios (OR), 95% confidence interval (CI), and p-values were reported. The regression model allowed us to isolate predictors of perceived needs for SRH information among in-school adolescents while considering variations in individual socio-demographic factors (age, gender, study groups, educational level, place of residence, work for pay, and socioeconomic status). The predictors of perceived SRH information needs of adolescents were assessed using the 6 outcome variables. Based on the mean score of 2.50 (maximum of 6 and minimum of 0), the outcome variable takes the value of “1” if an individual score is ≥ 50% and a value of “0” if an individual score is below 50%.

**Table 3:** Predictors of adolescents’ perceived need for SRH information

Variables	OR	p-value	95% CI
**Study group**			
Non-intervention group	1	Reference	Reference
Intervention group	3.13	< 0.001*	1.71–5.72
**Gender**			
Male	1	Reference	Reference
Female	1.33	0.253	0.81–2.19
**Age**	1.03	0.001*	1.01–1.04
**Educational Level**			
Junior Secondary	1	Reference	Reference
Senior Secondary	1.41	0.153	0.88–2.24
**Location of residence**			
Rural	1	Reference	Reference
Urban	0.31	< 0.001*	0.18–0.53
**Work for pay**			
No	1	Reference	Reference
Yes	1.09	0.802	0.57–2.08
**Socio-economic status**	1.10	0.394	0.88–1.38
**N**		500	

* Statistical significance (p-value < 0.05)

## Results

Table 4 shows the association of socio-demographic characteristics of the adolescents, across the study groups (intervention and non-intervention). A total of 266 students in the intervention group and 248 in the non-intervention group participated in the study. Majority of them were females, (72% in the intervention group and 64% in non-intervention group). About two-thirds of them were in senior secondary classes, (65% in intervention group and 60% in non-intervention group). There was no statistically significant difference between the two groups in all the socio-demographic variables.

**Table 4:** Association of socio-demographic characteristics of the adolescents across intervention and non-intervention

Variables (514)	Intervention group	Non-Intervention group	X^2^(p-value)
	Frequency (%) *n* = 266	Frequency (%) *n* = 248	
**Location of residence**			
Rural	136 (51.13)	126 (50.81)	0.10 (0.75)
Urban	130 (48.87)	122 (49.19)	
**Gender**			
Female	192 (72.18)	159 (64.11)	3.86 (0.05)
Male	74 (27.82)	89 (35.89)	
**Age group**			
Early adolescent	32 (12.03)	39 (15.73)	3.40 (0.18)
Middle adolescent	199 (74.81)	187 (75.40)	
Late adolescent	35 (13.16)	22 (8.87)	
**Level of schooling**			
Junior Secondary	93 (34.96)	99 (39.92)	1.35 (0.25)
Senior Secondary	173 (65.04)	149 (60.08)	
**Work for pay**			
No	214 (81.06)	206 (83.06)	0.35 (0.56)
Yes	50 (18.94)	42 (16.94)	
**Wealth index**	257(8.19)	245(8.19)	9.39(0.74)
Mean age (std. dev.)	15.60 (1.55)	15.29 (1.52)	2.31(0.97) ^

*Statistical significance (*p* < 0.05)

Table 5 shows the association of the various perceived SRH information needs of adolescents across intervention and non-intervention groups. Majority of the adolescents (82% of intervention group and 92% of non-intervention group) identified puberty and pubertal changes as perceived SRH information needs that should be provided to adolescents. This was found to be statistically significant (χ^2^ = 7.94; p-value = 0.01).

**Table 5:** Association of the various perceived SRH information needs of adolescents across intervention and non-intervention

Outcome	Intervention Frequency (%)	Non-Intervention Frequency (%)	X^2^(p-value)
Puberty and pubertal changes	185(82.22)	168(91.80)	7.94(0.01)*
Safe sex and sexual relations	127(56.44)	86(46.99)	3.61(0.06)
Use of condom	75(33.33)	55(30.05)	0.50(0.48)
Prevention of STIs/RTIs	74(32.89)	70(38.25)	1.27(0.26)
Sexual violence and rape	60(26.67)	41(22.40)	0.98(0.32)
Prevention of pregnancy and use of contraceptives	47(20.89)	32(17.49)	0.75(0.39)
N	225	183	

*Statistical significance (p-value < 0.05)

Table 3 shows the predictors of adolescent’s perceived need for SRH information. The table shows that Adolescents who received SRH intervention have 3.13 times the odds of perceiving the need for adolescents to be provided with SRH information than adolescents who did not receive SRH intervention. Put differently, the perception odds for SRH information are higher for the intervention group. The results in Table 3 also showed that the odds of perceiving the need for adolescents to be provided with SRH information for adolescents who reside in urban communities are 0.31 times the odds for adolescents who resides in rural communities. That is, the perception odds are higher adolescents who reside in rural communities.

Tables 1 and 2 show the logistic regression of adolescents’ perceived need for specific SRH information that should be provided.

The results in Table 1 showed that the odds of perceiving the need for adolescents to be provided with information on puberty and pubertal changes, safe sex and sexual relations, and prevention of pregnancy and use of contraceptives for adolescents who reside in urban communities are 0.30, 0.33, and 0.28 times the odds for adolescents who resides in rural communities, respectively.

The results in Table 2 showed adolescents in senior secondary school have 2.21 times the odds of perceiving the need for adolescents to be provided with specific SRH information than adolescents who are in junior secondary school.

## Discussion

This study utilized a quantitative method to assess the sexual and reproductive health information needs of adolescent boys and girls in public secondary schools. The study findings showed that most adolescents in both intervention and non-intervention groups perceived the need for them to be provided with information about puberty and pubertal changes. Many of them reported the need for adolescents to be provided with information about safe sex and sexual relations while a considerable number of adolescents in this study pointed out the need for information about condom use and prevention of STI/RTIs to be provided. Conforming to these findings, adolescents and adult stakeholders in a qualitative study perceived the need for adolescents to be provided with continuous sexuality education [26]. A previous study revealed that adolescents and young people reported that they are not receiving all the right information they need to make informed choices regarding their SRH [27].

This study showed that some demographic factors such as (location, and age) and receiving SRH intervention were predictors of adolescents’ perceived need for SRH information. These predictors contribute greatly to adolescents’ perceived need for SRH information which could either hinder or promote adolescents’ access to or utilization of SRH information and services. Slightly similar to our findings, Okeke and colleagues revealed that the SRH need of adolescents varies based on some social stratifies such as gender, marital and schooling status [26]. Considering several factors, studies have shown that all adolescents should be empowered with the right SRH information through regular information campaigns, seminars, workshops, and conferences [26, 28]. This will create demand for SRH information and other services among adolescents, empowering them to overcome their SRH needs [26].

The findings from our study show that adolescents who live in urban communities were less likely to perceive the need for information on the prevention of pregnancy and the use of contraceptives. A study carried out by Ezenwaka et al. revealed that the use of contraceptives is largely limited in Ebonyi state due to a lack of awareness, poor knowledge of contraceptives, fear of side effects, culture/religion, and societal shaming [20]. Information on the prevention of pregnancy and the use of contraceptives remains a common SRH need among adolescents after interventions have been implemented in Ebonyi state hence; there is a need for continuous intervention to ensure that these needs are addressed among adolescents in schools. Also in our study, adolescents in the urban area were less likely to perceive the need to be provided with information about puberty and pubertal change, sexual relations and safe sex, use of condoms, sexual violence/rape, and prevention of STIs/RTIs. Previous investigations [19, 29–31], stated that adolescents who access health facilities for SRH information and other services were mostly urban dwellers. Hence, rural dwellers were less likely to access health facilities for SRH information and they are most likely to need information about sexual and reproductive health matters. However, the finding from these studies was inconsistent with our study which could be due to contextual differences.

From our study, adolescents in the intervention group were more likely to have perceived the need for SRH information than those in the non-intervention group in general. Specifically, adolescents in the intervention group were more likely to perceive safe sex and sexual relation, prevention of pregnancy and the use of contraceptives, use of condoms, and sexual violence and rape as SRH information needs, when compared to adolescents in the non-intervention group. Interventions implemented to address SRH have previously been shown to be effective in improving the SRH needs of adolescents [29, 32–34]. However, it is important to mention that SRH information needs on puberty and pubertal changes and prevention of STIs/RTIs were higher among non-intervention group. The reason for the observed differences in the level of perceived SRH information needs by topics across intervention and non-intervention groups could be due to the intervention, which create awareness about SRH among adolescents. This is not likely the only reason driving the differences as since information needs on puberty and pubertal changes and prevention of STIs/RTIs are higher among non-intervention group. Individual unobserved differences that have been formed over time may also driving the observed differences.

Adolescent’s educational level was statistically associated with condom use, as senior secondary students were approximately 2 times more likely to perceive the need for information about condom use among adolescents. In agreement with this study which reveals that senior secondary students were perceived to have higher needs for SRH information, previous studies [30, 35–38] have shown that adolescents at higher levels of education are more likely to access SRH information and services. Therefore, adolescents who are in senior secondary classes are more likely to need information on condom use than adolescents in junior secondary classes.

The finding of an association between age and perceived SRH information needs is consistent with a study carried out by Abajobir et al., who reported that age is significantly associated with the demand for SRH information and services [35]. From our study, older adolescents were more likely to perceive puberty and pubertal changes, sexual relations and safe sex, prevention of pregnancy, and use of contraceptives as SRH information needs. An earlier study revealed that the need for SRH information among adolescents and young people increases as they become older [27]. In the earlier study, adolescents and young people reported that the provision of SRH information should be continuous throughout their developmental stages [27]. Information should be tailored to meet the SRH needs of each adolescent category.

The engagement of both adolescents in schools where SRH intervention have been implemented and those in non-intervention schools enables better representation of data from these two categories on their perceived SRH information need. This study is, however, limited in that it reports findings from only in-school adolescent boys and girls aged 13 to 18 years. The study presents data on the specific SRH information delivered to adolescents in secondary school. There is also the issue of social desirability when it concerns adolescents and sexuality issues, hence the likelihood of introducing bias. Therefore, its generalizability should be applied with caution. Furthermore, the study made use of only a quantitative research approach to identify the adolescent’s perceived SRH information needs. Future research should incorporate an in-depth exploration of these issues through qualitative research methods for a better understanding of these views.

## Conclusion

This study showed that adolescents’ perceived need for SRH information differed based on their socio-demographics. Adolescents’ age, location of residence, and study group were found to be strong predictors of SRH information needs. Adolescents who received the intervention were more likely to perceive the need to provide them with SRH information such as safe sex and sexual relations, prevention of pregnancy, and use of contraceptives. Also, adolescents in senior secondary were likely to perceive the need for information on how to use condoms.

The implementation of strategic SRH intervention is effective in addressing adolescent SRH information needs. Therefore, to adequately address adolescents’ SRH information needs, the continuous implementation of strategic SRH interventions with consideration of the social and demographic predictors of SRH information identified are required by health professionals, researchers, program implementers, and policymakers. Also, there is a need for the provision and incorporation of these identified SRH information needs to the school educational curriculum in Ebonyi State Nigeria. Further studies need to be done on the certain (type) SRH information delivered to adolescents in school, in Ebonyi state Nigeria.

### Electronic supplementary material

Below is the link to the electronic supplementary material.

Supplementary Material 1

Supplementary Material 2

Supplementary Material 3

## Data Availability

The analyzed data presented in this study is a subset of datasets generated from a large project and the datasets are not publicly available due to our organizational policies/guidelines on funded implementation research projects. However, the project datasets will be made available by the corresponding author on reasonable request.

## Abbreviations

SRH: Sexual and Reproductive Health
HIV/AIDS: Human immunodeficiency virus/acquired immunodeficiency syndrome
STIs: Sexually Transmitted Infections
RTIs: Reproductive Tract Infection
