# Supplementary material for: Sexual and reproductive health information needs; an inquiry from the lens of in-school adolescents in Ebonyi State, Southeast Nigeria
Source: BMC Public Health. 2024 Apr 22;24:1105. doi: 10.1186/s12889-024-18584-w (PMC11034149; doi:10.1186/s12889-024-18584-w)
Supplement: Supplementary file 1 — Supplementary Material 1 [file 12889_2024_18584_MOESM1_ESM.docx]

Table S1: Description of selected schools in terms of location, educational system, intervention, and number of surveyed students

|  | Location  (Urban or rural) | Education system  (co-ed; boys; girls) | School-based intervention^1^ (Yes/No) | No. of students surveyed |
| --- | --- | --- | --- | --- |
| School 1 | Urban | Girls | Yes | 44 |
| School 2 | Rural | Co-ed | Yes | 51 |
| School 3 | Rural | Co-ed | Yes | 42 |
| School 4 | Urban | Co-ed | Yes | 43 |
| School 5 | Rural | Co-ed | Yes | 43 |
| School 6 | Urban | Girls | Yes | 43 |
| School 7 | Urban | Co-ed | No | 40 |
| School 8 | Rural | Co-ed | No | 41 |
| School 9 | Rural | Co-ed | No | 42 |
| School 10 | Urban | Co-ed | No | 40 |
| School 11 | Rural | Co-ed | No | 43 |
| School 12 | Urban | Co-ed | No | 42 |
