# Supplementary material for: Sexual and reproductive health information needs; an inquiry from the lens of in-school adolescents in Ebonyi State, Southeast Nigeria
Source: BMC Public Health. 2024 Apr 22;24:1105. doi: 10.1186/s12889-024-18584-w (PMC11034149; doi:10.1186/s12889-024-18584-w)
Supplement: Supplementary file 2 — Supplementary Material 2 [file 12889_2024_18584_MOESM2_ESM.docx]

Table A1. Summary statistics of the outcome variable (perception composite score)

| Variable | Observations | Mean | Standard Deviation | Minimum | Maximum |
| --- | --- | --- | --- | --- | --- |
| Perception score | 408 | 4.1 | 2.6 | 0 | 10 |

Table A2. Distribution of perception score (intervention and non-intervention groups)

| Group | Mean |
| --- | --- |
| Intervention group | 4.3 |
| Non-intervention group | 3.9 |
