# Supplementary material for: Sexual and reproductive health information needs; an inquiry from the lens of in-school adolescents in Ebonyi State, Southeast Nigeria
Source: BMC Public Health. 2024 Apr 22;24:1105. doi: 10.1186/s12889-024-18584-w (PMC11034149; doi:10.1186/s12889-024-18584-w)
Supplement: Supplementary file 3 — Supplementary Material 3 [file 12889_2024_18584_MOESM3_ESM.docx]

**Unadjusted logistic regressions**

**Table B1.
Logistic regression**

| Puberty_pubertal change | Coef. | | St.Err. | t-value | | p-value | [95% Conf | | Interval] | | Sig |
| --- | --- | --- | --- | --- | --- | --- | --- | --- | --- | --- | --- |
| Intervention group | .413 | | .133 | -2.76 | | .006 | .22 | | .775 | | *** |
| Constant | 11.2 | | 3.018 | 8.97 | | 0 | 6.604 | | 18.993 | | *** |
|  | | | | | | | | | | | |
| Mean dependent var | | 0.865 | | | SD dependent var | | | 0.342 | |  |  |
| Pseudo r-squared | | 0.026 | | | Number of obs | | | 408 | |  |  |
| Chi-square | | 8.279 | | | Prob > chi2 | | | 0.004 | |  |  |
| Akaike crit. (AIC) | | 318.382 | | | Bayesian crit. (BIC) | | | 326.404 | |  |  |
| **** p<.01, ** p<.05, * p<.1* | | | | | | | | | | | |
|  | | | | | | | | | | | |

**Table B2.
Logistic regression**

| safe_sex_sexual_re~n | Coef. | | St.Err. | t-value | | p-value | [95% Conf | | Interval] | | Sig |
| --- | --- | --- | --- | --- | --- | --- | --- | --- | --- | --- | --- |
| Intervention group | 1.462 | | .292 | 1.90 | | .058 | .988 | | 2.163 | | * |
| Constant | .887 | | .131 | -0.81 | | .416 | .663 | | 1.185 | |  |
|  | | | | | | | | | | | |
| Mean dependent var | | 0.522 | | | SD dependent var | | | 0.500 | |  |  |
| Pseudo r-squared | | 0.006 | | | Number of obs | | | 408 | |  |  |
| Chi-square | | 3.615 | | | Prob > chi2 | | | 0.057 | |  |  |
| Akaike crit. (AIC) | | 565.198 | | | Bayesian crit. (BIC) | | | 573.221 | |  |  |
| **** p<.01, ** p<.05, * p<.1* | | | | | | | | | | | |
|  | | | | | | | | | | | |

**Table B3.
Logistic regression**

| prevntion_prevention | Coef. | | St.Err. | t-value | | p-value | [95% Conf | | Interval] | | Sig |
| --- | --- | --- | --- | --- | --- | --- | --- | --- | --- | --- | --- |
| Intervention group | 1.246 | | .317 | 0.86 | | .388 | .757 | | 2.052 | |  |
| Constant | .212 | | .041 | -7.97 | | 0 | .145 | | .31 | | *** |
|  | | | | | | | | | | | |
| Mean dependent var | | 0.194 | | | SD dependent var | | | 0.396 | |  |  |
| Pseudo r-squared | | 0.002 | | | Number of obs | | | 408 | |  |  |
| Chi-square | | 0.753 | | | Prob > chi2 | | | 0.386 | |  |  |
| Akaike crit. (AIC) | | 404.263 | | | Bayesian crit. (BIC) | | | 412.285 | |  |  |
| **** p<.01, ** p<.05, * p<.1* | | | | | | | | | | | |
|  | | | | | | | | | | | |

**Table B4
Logistic regression**

| condom_use | Coef. | | St.Err. | t-value | | p-value | [95% Conf | | Interval] | | Sig |
| --- | --- | --- | --- | --- | --- | --- | --- | --- | --- | --- | --- |
| Intervention group | 1.164 | | .25 | 0.71 | | .48 | .764 | | 1.772 | |  |
| Constant | .43 | | .069 | -5.24 | | 0 | .313 | | .589 | | *** |
|  | | | | | | | | | | | |
| Mean dependent var | | 0.319 | | | SD dependent var | | | 0.467 | |  |  |
| Pseudo r-squared | | 0.001 | | | Number of obs | | | 408 | |  |  |
| Chi-square | | 0.501 | | | Prob > chi2 | | | 0.479 | |  |  |
| Akaike crit. (AIC) | | 514.177 | | | Bayesian crit. (BIC) | | | 522.199 | |  |  |
| **** p<.01, ** p<.05, * p<.1* | | | | | | | | | | | |
|  | | | | | | | | | | | |

**Table B5.
Logistic regression**

| sexual_violence | Coef. | | St.Err. | t-value | | p-value | [95% Conf | | Interval] | | Sig |
| --- | --- | --- | --- | --- | --- | --- | --- | --- | --- | --- | --- |
| Intervention group | 1.259 | | .293 | 0.99 | | .322 | .798 | | 1.987 | |  |
| Constant | .289 | | .051 | -7.01 | | 0 | .204 | | .409 | | *** |
|  | | | | | | | | | | | |
| Mean dependent var | | 0.248 | | | SD dependent var | | | 0.432 | |  |  |
| Pseudo r-squared | | 0.002 | | | Number of obs | | | 408 | |  |  |
| Chi-square | | 0.989 | | | Prob > chi2 | | | 0.320 | |  |  |
| Akaike crit. (AIC) | | 459.666 | | | Bayesian crit. (BIC) | | | 467.688 | |  |  |
| **** p<.01, ** p<.05, * p<.1* | | | | | | | | | | | |
|  | | | | | | | | | | | |

**Table B6.
Logistic regression**

| prevention_sti | Coef. | | St.Err. | t-value | | p-value | [95% Conf | | Interval] | | Sig |
| --- | --- | --- | --- | --- | --- | --- | --- | --- | --- | --- | --- |
| Intervention group | .791 | | .165 | -1.13 | | .26 | .526 | | 1.189 | |  |
| Constant | .619 | | .094 | -3.15 | | .002 | .46 | | .835 | | *** |
|  | | | | | | | | | | | |
| Mean dependent var | | 0.353 | | | SD dependent var | | | 0.478 | |  |  |
| Pseudo r-squared | | 0.002 | | | Number of obs | | | 408 | |  |  |
| Chi-square | | 1.269 | | | Prob > chi2 | | | 0.260 | |  |  |
| Akaike crit. (AIC) | | 532.518 | | | Bayesian crit. (BIC) | | | 540.541 | |  |  |
| **** p<.01, ** p<.05, * p<.1* | | | | | | | | | | | |
|  | | | | | | | | | | | |
